# Prevention of In‐Stent Restenosis After PCI by Saponin Natural Products: Inhibition of Platelet Activation

**DOI:** 10.1111/jcmm.71056

**Published:** 2026-02-12

**Authors:** Xueli Lei, Chunlei Lv, Yu Zhang, Zhiyu Li, Hao Shen, Li Zhu

**Affiliations:** ^1^ Department of Cardiology Tai'an 88 Hospital Taian Shandong China; ^2^ Department of Laboratory Medicine Wujiang Branch of Suzhou Central Blood Station Suzhou China; ^3^ Department of Laboratory Medicine Suzhou Ninth Hospital Affiliated to Soochow University Suzhou Jiangsu China

**Keywords:** antithrombotic therapy, in‐stent restenosis, natural products, platelet activation, saponins

## Abstract

Percutaneous coronary intervention (PCI) enables coronary revascularisation and restores haemodynamic stability but also carries risks of delayed complications including in‐stent restenosis (ISR), a chronic progressive disease with endovascular damage after PCI, which compromises the long‐term efficacy of PCI. Currently, the main treatment strategies for ISR include interventional and drug therapies. From the era of using aspirin as a single antiplatelet agent to the ‘gold standard’ era of dual antiplatelet therapy, the risk of ISR after PCI has been reduced. However, long‐term use of antiplatelet drugs inevitably causes a series of side effects such as gastrointestinal mucosal damage and bleeding, which have become key limiting factors in clinical treatment. Saponin natural products have been used to mitigate ISR progression by targeting platelet dysfunction. Specifically, these compounds directly bind to platelet membrane receptors, block ligand–receptor interactions, inhibit the secretion of α‐granule and dense‐granule contents, regulate intracellular signalling pathways and platelet metabolism, inhibit release of inflammatory mediators, and suppress platelet aggregation. This article reviews the progresses on the application of the active components in saponin natural products to inhibit platelet activation in the development of ISR after PCI, aiming to provide a superior approach for the comprehensive treatment of ISR after PCI.

## 1 Introduction

Cardiovascular disease (CVD) is one of the leading causes of death and disability worldwide. With economic development, aging and lifestyle changes, the incidence rate and mortality of CVD are increasing year by year and the onset tends to be younger, which has brought an increasing economic burden to residents and society and has become a major public health problem [1]. Percutaneous coronary intervention (PCI), as an effective method of revascularisation, reduces the incidence and mortality of cardiovascular events such as acute myocardial infarction. However, the complication of in‐stent restenosis (ISR) after PCI remains an urgent clinical issue that needs to be addressed [2]. Studies indicate that during the early era of bare metal stents (BMS), the incidence of ISR was relatively high, ranging from 20% to 30% [3]. With the widespread adoption of drug‐eluting stents (DES), the ISR rate remains 5%–10% [4]. Although the incidence rate of ISR has decreased to some extent, it remains an important factor affecting the long‐term efficacy and prognosis of PCI.

The essential nature of ISR is a chronic progressive pathological process characterised by vascular endothelial injury following PCI, which subsequently triggers neointimal tissue hyperplasia. Stent implantation after PCI causes endothelial damage by directly triggering platelet activation, which may play a key role in the occurrence and development of ISR by disrupting the structural integrity and stability of the vascular wall. Platelet activation is a key regulatory link in the pathological process of ISR, which plays a role throughout the entire process from vascular injury to intimal hyperplasia. Platelet activation, adhesion and aggregation on the stent metal surface or exposed vascular wall components leads to thrombotic vessel occlusion [5]. At present, antiplatelet therapy after PCI is the basic treatment and a key measure to prevent postoperative thrombosis and reduce the risk of cardiovascular adverse events, which is crucial for maintaining vascular patency. The treatment methods for ISR mainly include interventional therapy (re‐stenting, cutting balloon technique, laser angioplasty, etc.), drug therapy (antiplatelet therapy, statins, ACEI/ARB, etc.); however, there has been a major dissatisfaction over the long‐term. For example, long‐term use of antiplatelet drugs inevitably brings a series of side effects such as gastrointestinal mucosal damage and bleeding, which is required for a careful evaluation, management and individualised treatment in clinical practice. In the past decade, many studies have shown that saponin natural products have the potential to exert antithrombotic effects by regulating platelet function, which can indirectly slow down the occurrence and development of ISR. This review focuses on the progress of using saponin natural products to regulate platelet function for the prevention and treatment of ISR.

## 2 The Preventive Effects of Saponin Natural Products on Coronary Heart Diseases

**FIGURE 1:**
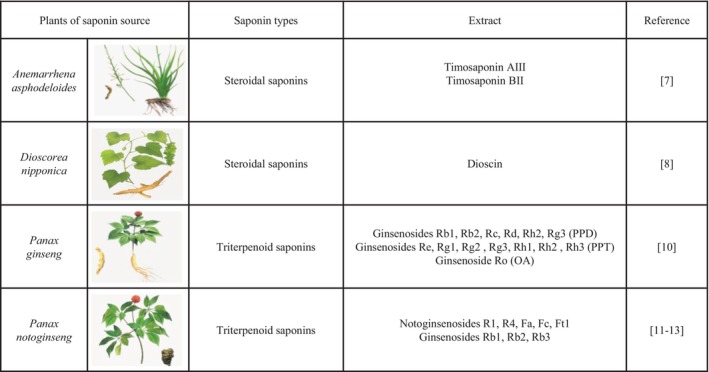
Types of saponin natural products. Anemarrhena *asphodeloides* and *Dioscorea nipponica* belong to steroidal saponins, with main extracts including Timosaponin AIII, Timosaponin BII and Dioscin (Rows 2–3). *Panax ginseng* and *Panax notoginseng* belong to triterpenoid saponins, with main extracts including Ginsenosides Rb1, Rb2, Rc, Rd, Rh2, Rg3 (PPD), Ginsenosides Re, Rg1, Rg2, Rg3, Rh1, Rh2, Rh3 (PPT), Ginsenoside Ro (OA), Notoginsenosides R1, R4, Fa, Fc, Ft1 and Ginsenosides Rb1, Rb2, Rb3 (Rows 4–5).

**FIGURE 2:**
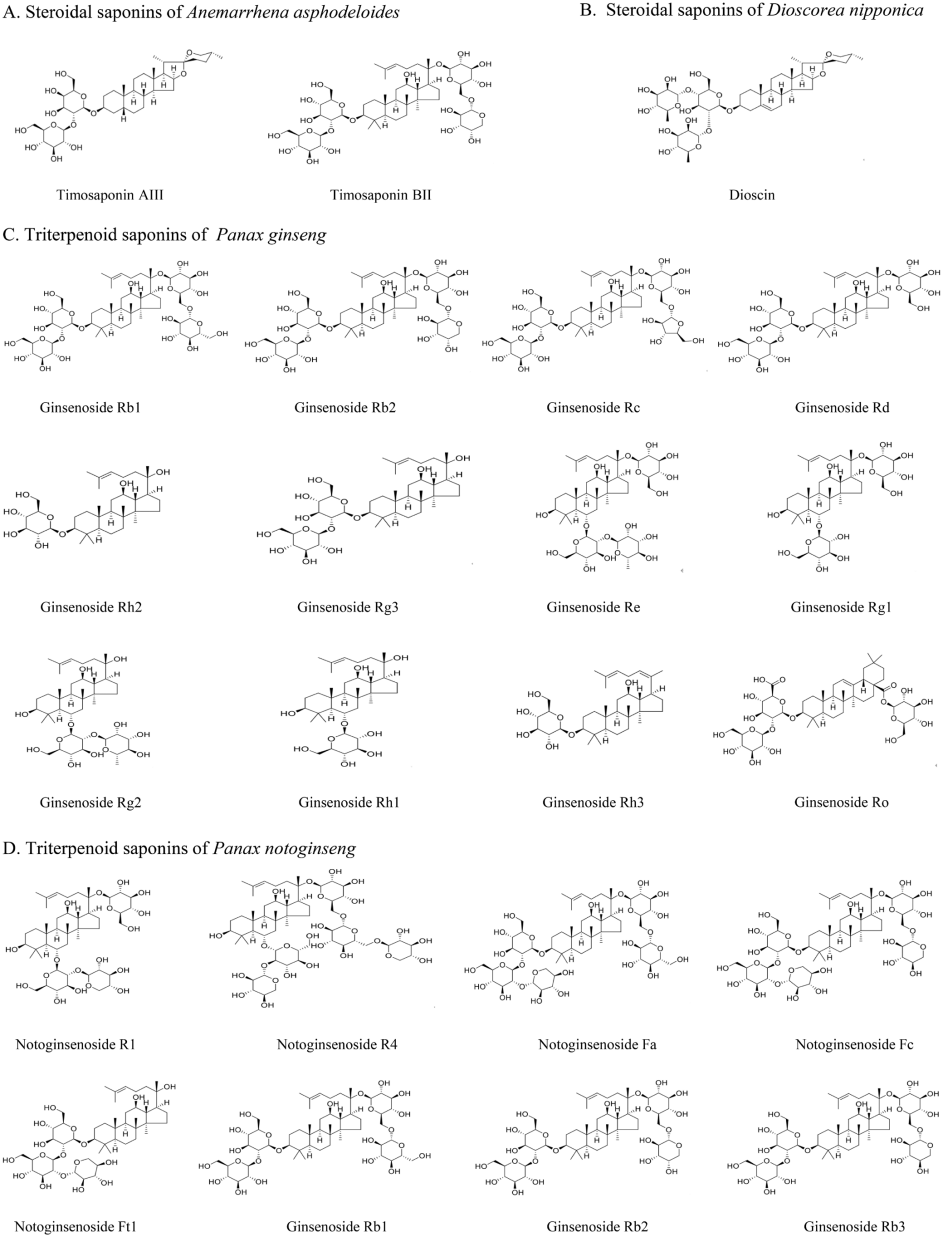
Chemical structure of saponin natural products. The basic skeleton of steroidal saponins is centred on a 27‐carbon steroidal aglycone with one or more oligosaccharide chains covalently linked via a glycosidic bond (A, B). The core skeleton structure of triterpenoid saponins consists of a C_30_ triterpene aglycone and one or more oligosaccharide chains covalently linked via glycosidic bonds (C, D).

Saponin natural products are a class of important secondary metabolites widely present in plants, animals and marine organisms. They are naturally extracted from plants, either as mixtures or multiple monomers, and have been an important source of drugs for treating various diseases in humans [6]. As illustrated in Figure 1, the bioactive saponins of *Anemarrhena asphodeloides* and *Dioscorea nipponica* species are steroidal saponins. The extract of *Anemarrhena asphodeloides* is obtained from the dried rhizomes of *Anemarrhena asphodeloides* Bge. (Liliaceae), mainly containing Timosaponin AIII and Timosaponin BII [7]. The extract of *Dioscorea nipponica* species is derived from the dried rhizomes of plants in the genus *Dioscorea* (Dioscoreaceae), with Dioscin as the major component [8]. The basic skeleton of steroidal saponins is centred on a 27‐carbon steroidal aglycone with one or more oligosaccharide chains covalently linked via a glycosidic bond [9] (Figure 2A,B). The bioactive saponins of *Panax ginseng* and *Panax notoginseng* are triterpenoid saponins. The extract of *Panax ginseng* , isolated from the dried roots and rhizomes of *Panax ginseng* C.A. Mey. (Araliaceae), contains ginsenosides that are classified into three categories: protopanaxadiol type (PPD‐type, e.g., Ginsenosides Rb1, Rb2, Rc, Rd., Rh2, Rg3), protopanaxatriol type (PPT‐type, e.g., Ginsenosides Re, Rg1, Rg2, Rg3, Rh1, Rh2, Rh3) and oleanolic acid type (OA‐type, e.g., Ginsenoside Ro) [10]. The extract of *Panax notoginseng* is obtained from the dried roots and rhizomes of *Panax notoginseng* (Burk.) F.H. Chen (Araliaceae), primarily including Notoginsenosides R1, R4, Fa, Fc, Ft1 and Ginsenosides Rb1, Rb2, Rb3 [11, 12, 13]. The core skeleton structure of triterpenoid saponins consists of a C_30_ triterpene aglycone and one or more oligosaccharide chains, wherein the triterpene aglycone is a tetracyclic or pentacyclic terpenoid structure composed of 30 carbon atoms, while the oligosaccharide chains are covalently linked to the hydroxyl or carboxyl groups of the aglycone via glycosidic bonds [14, 15] (Figure 2C,D).

Saponin natural products constitute a significant source of novel antithrombotic agents. Following isolation, purification and structural characterisation, their bioactive components demonstrate multifaceted pharmacological activities, including antiplatelet aggregation, anti‐inflammatory and antioxidant effects, metabolic regulation, antitumor activity and central nervous system modulation [16, 17, 18, 19, 20, 21, 22, 23, 24, 25, 26, 27]. An increasing number of studies on antiplatelet and anticoagulant activities are using extracts or compounds of saponin natural products, especially in the field of coronary heart disease (CHD), which have broad application prospects (Table 1).

**TABLE 1:** Saponin natural products for the treatment of coronary heart disease.

Coronary heart disease	Ingredients—dosage	Experimental models	Mechanisms	References
Myocardial ischemia–reperfusion injury	Ginseng saponin Rh3 4, 8, 16 mg/(kg day)	Rat model of ischemia–reperfusion injury	↑Bcl‐2, ↓Bax	[28]
	Ginsenoside Re 135 mg/kg	A rat model of myocardial infarction	↑FAK, ↑PI3K‐p110α, ↑Akt	[29]
	Timosaponin BII 50 mg/kg, 100 mg/kg	A rat model of myocardial infarction	↓SOD, ↑MDA, ↑Nrf‐2	[30]
	Timosaponin BII 921.07 g/mol	In vivo models of isoproterenol‐induced myocardial injury	↓H_2_O_2_‐induced H9c2	[31]
	Astragaloside IV 250 μg/mL	A murine model of coxsackievirus B3‐induced viral myocarditis	↓CK‐MB, ↓LDH, ↓IFN‐γ, ↓IL‐6, ↓FAS, ↓FASL, ↓cleaved caspase‐8, ↓cleaved caspase‐3	[32]

CHD, as the primary cause of myocardial ischemia, carries a critical therapeutic paradox: reperfusion strategies, while essential, may trigger myocardial ischemia–reperfusion injury (MIRI), leading to secondary cardiomyocyte damage or even necrosis. Within this context, triterpenoid saponins demonstrate significant potential for preventing and treating MIRI [33]. Ginsenoside Rh3 can promote the reduction of oxygen free radicals, upregulate the expression of cardiomyocyte related apoptosis protein Bcl‐2, and inhibit the expression of Bax protein, thereby exerting a significant protective effect on myocardial injury in ischemia–reperfusion rats [28]. The protective effect and mechanism of Ginsenoside Re on cardiac function and left ventricular remodelling in rats with myocardial infarction may be achieved by regulating the AMPK/TGF‐β1/Smad2/3 and FAK/PI3K‐p110α/Akt signalling pathways, improving cardiac dysfunction caused by myocardial ischemia and reducing ventricular remodelling [29]. Some studies have also shown that Timosaponin BII mainly inhibits inflammatory cytokines, increases the expression of heme oxygenase isoform 1 (HO‐1) and nuclear respiratory factor 2 (Nrf‐2), and enhances antioxidant capacity to reduce isoproterenol induced rat myocardial cell apoptosis and exert a protective effect against ischemic heart disease such as myocardial infarction [30]. In vitro, it can prevent hydrogen peroxide induced H9c2 myocardial cell damage through the PI3K/Akt pathway [31]. Coxsackievirus infection is a major contributor to myocardial injury‐related diseases such as viral myocarditis. Studies have shown that Astragaloside IV has anti‐coxsackievirus activity. Inhibiting the FAS/FASL signalling pathway significantly reduces the serum levels of creatine kinase‐MB (CK‐MB) and lactate dehydrogenase (LDH), thereby suppressing coxsackievirus B3‐induced cardiomyocyte apoptosis and alleviating virus‐induced myocardial damage and fibrosis [32].

## 3 The Key Mechanism of ISR After PCI

PCI is a minimally invasive surgical method used to treat CHD, in which instruments such as balloon catheters or stents are inserted into the site of coronary artery stenosis using cardiac catheterisation technology to dilate the narrowed blood vessels and restore myocardial blood supply. ISR following PCI is a complex, multifactorial and multistage process. It involves multiple interconnected mechanisms, including vascular injury, inflammatory responses and cellular proliferation/migration. Concurrently, it engages the activation of platelets and the activation of the coagulation‐fibrinolytic system, reflecting the intricate interplay among multiple biological systems.

Endothelial injury induced by balloon dilation and stent implantation is universally recognised as the initiating event in the pathological cascade leading to ISR. During PCI, mechanical trauma from balloon inflation and stent deployment triggers a cascade of vascular wall reparative responses. This includes proliferation and migration of vascular smooth muscle cells (VSMCs), coupled with increased synthesis of extracellular matrix (ECM). While these repair mechanisms facilitate vascular healing, they paradoxically predispose to luminal renarrowing [34, 35]. Endothelial cell proliferation and migration form a protective neointimal layer over the stent, which is crucial for mitigating restenosis. However, in clinical reality, many PCI patients exhibit comorbidities, such as hypertension, diabetes or dyslipidemia. These conditions impair mobilisation of endothelial progenitor cells while simultaneously enhancing VSMCs' proliferative capacity. Consequently, endothelial regeneration remains incomplete, leaving segments of the stent exposed and perpetuating the risk of pathological remodelling.

Inflammatory response is one of the core mechanisms underlying ISR and runs through the entire process of ISR. Regardless of the type of stent implanted in the coronary artery, as a foreign body, it will cause sustained mechanical damage to the vascular wall and trigger an inflammatory response in the body. Multiple inflammatory cells are chemotactic and infiltrate target blood vessels, synthesising and releasing inflammatory factors, such as IL‐1, IL‐6, IL‐8, MMP, TNF‐α, etc. These factors act on endothelial cells, liver cells, platelets, etc., promoting their secretion of cytokines such as endothelin (ET), ICAM, VCAM, CRP, PDGF, VEGF, etc. ET constricts blood vessels, ICAM and VCAM facilitate leukocyte adhesion and migration, CRP reflects the degree of inflammation, and PDGF and VEGF stimulate VSMCs [36, 37]. Under the synergy of inflammatory factors and cytokines, VSMCs transform into a synthetic phenotype, proliferate and migrate towards the endometrium, and synthesise a large amount of ECM. At the same time, platelet activation and aggregation form thrombosis, ultimately leading to ISR.

It is worth noting that platelet activation is the core ‘engine’ driving the occurrence and development of ISR. It runs through the entire process of ISR and promotes pathological progression, from triggering platelet adhesion and aggregation to form thrombus ‘outpost’ in vascular endothelial injury, to mediating inflammatory reactions and causing neointimal hyperplasia ‘storms’ in VSMCs proliferation. Under normal circumstances, platelets cannot adhere to the surface of endothelial cells. After PCI, the endothelial structure and function are damaged, and the subendothelial collagen fibres are exposed. The von Willebrand factor (vWF) in the plasma binds to collagen fibres, causing conformational changes in vWF. The allosteric vWF binds to GPIb on the platelet membrane, promoting platelet adhesion and releasing large amounts of platelet aggregating agents such as ADP, vWF, 5‐HT, Ca^2+^, fibrinogen, thromboxane A_2_ (TXA_2_), etc. Under the latter action, integrins αIIbβ3, also called GPIIb/GPIIIa complex, are exposed and bind to fibrinogen, thereby promoting more platelet aggregation and ultimately forming platelet thrombus. Considering the role of platelet activation in the formation of ISR, higher platelet activity may be a poor prognostic factor for ISR patients. Therefore, antiplatelet therapy can effectively maintain vascular patency, reduce the incidence of ISR and thrombotic occlusion, ensure blood supply to important organs such as myocardium and brain tissue, and is a key treatment for ISR patients [38].

## 4 Molecular Mechanism and Efficacy of Platelet Targeted ISR Prevention

**FIGURE 3:**
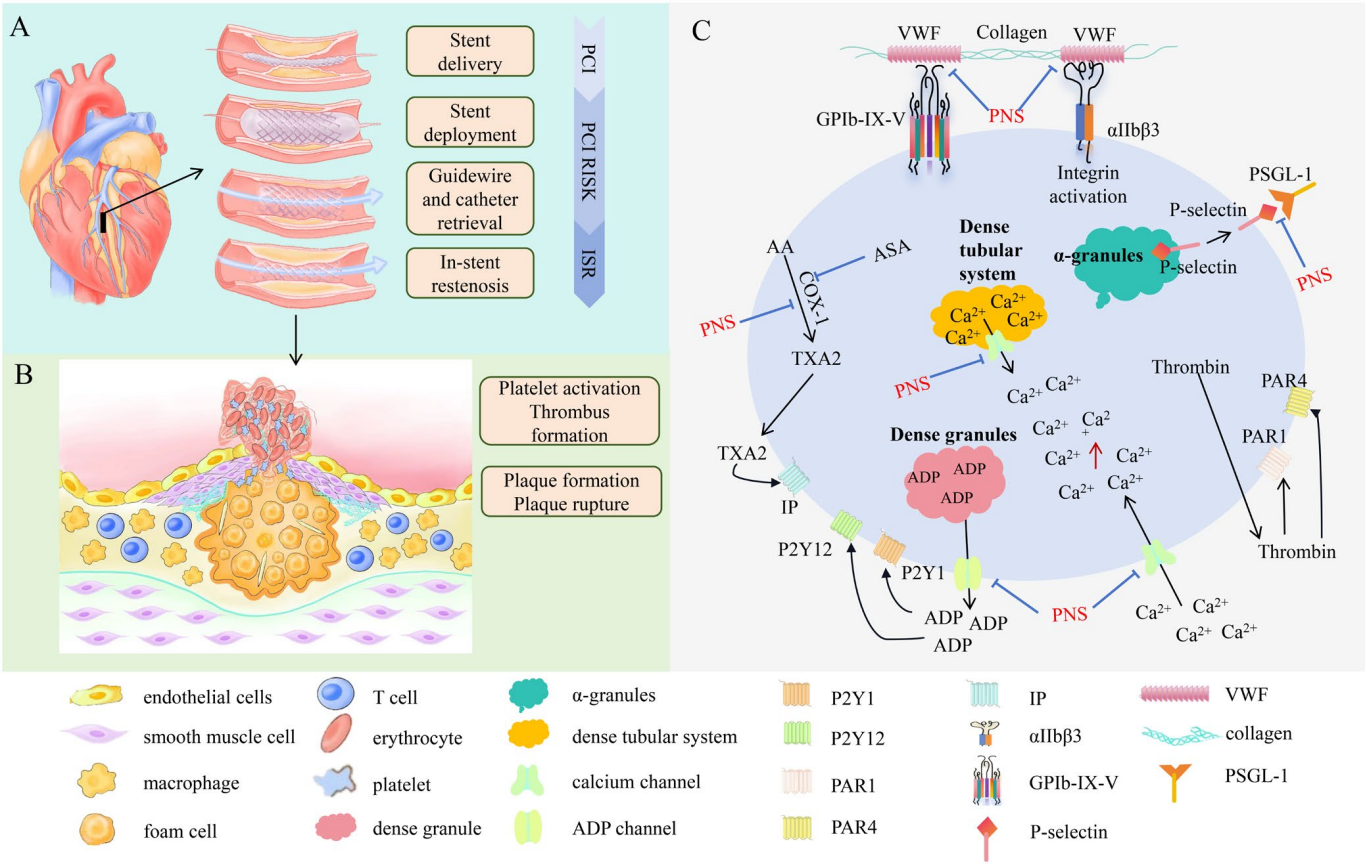
Application of saponin natural products to prevent the development of ISR after PCI during treatment of myocardial infarction by inhibiting platelet activation. (A) The development of ISR after PCI. PCI is performed on the coronary arteries to provide oxygen to the myocardium. The stent with balloon is inserted into artery. After the balloon inflated, the stent is expanded. After the balloon is deflated and removed, the guide wire will be withdrawn, and the stent remains. However, there is a risk of ISR occurring months to years after PCI. (B) Plaque rupture, platelet activation and thrombus formation. Plaque rupture leads to platelet aggregation and activation, which in turn promotes thrombus formation and causes ISR complications, which to some extent limits the long‐term efficacy of PCI. (C) ISR prevention by saponin natural products targeting platelet activation. Saponin natural products directly bind to platelet membrane receptors, block ligand–receptor interactions, inhibit the secretion of α‐granule and dense‐granule contents, regulate intracellular signalling pathways and platelet metabolism, inhibit release of inflammatory mediators, and suppress platelet aggregation. AA, arachidonic acid; COX‐1, cyclooxygenase‐1; IP, prostanoid receptor; PAR, Protease‐activated receptor; PSGL‐1, P‐selectin glycoprotein ligand 1; TXA_2_, thromboxane A2; vWF, von Willebrand factor.

Platelets are anucleate cells resulting from the fragmentation of megakaryocytes and play a central role in physiological haemostasis and pathological thrombosis. The core pathological mechanism of ISR involves the cascade reaction of thrombotic inflammation caused by abnormal platelet activation after vascular injury [39]. In recent years, studies have found that saponin natural products can effectively intervene in ISR by regulating platelet function, especially targeting its membrane glycoprotein receptors, adhesion molecules, particle release and inflammatory mediators. The regulation of platelet function is of great significance in the occurrence and development of ISR, and its molecular mechanisms mainly include the following aspects (Figure 3 and Table 2).

**TABLE 2:** Molecular mechanism and efficacy of platelet targeted ISR prevention.

Pathways	Ingredients—dosage	Experimental models	Mechanisms	References
Interact with platelet membrane glycoprotein receptors	Ginsenoside Ro 50–300 μM	Thrombin‐induced human platelet aggregation	↓Ca^2+^, ↑cAMP	[40]
Inhibit secretion of platelet adhesion receptor P‐selectin	*Panax notoginseng* saponins 3 g/day	Platelet aggregation induced by COL, EPI, ADP and AA as aggregation‐inducing agents	↓vWF: Ag, ↓P‐selectin, ↓MAR, ↑TT, ↑APTT, ↑PT	[41]
Reduce the release of platelet particles	*Panax notoginseng* saponins Rb1 0.01–10 μmol/L	Rat myocardial hypertrophy model	↓Na^+^‐K^+^‐ATP, ↓Ca^2+^	[42]
	*Panax notoginseng* extract 500 mg/kg	Collagen‐induced platelet aggregation model of rat	↓Ca^2+^, ↓Fibrinogen	[43]
Inhibit platelet metabolic pathways	*Panax notoginseng* saponins 118.8 mg/(kg·day)	The AMI model of Wistar rat	↓COX‐1, ↓TXB_2_	[44]
Inhibit release of inflammatory mediators	*Panax notoginseng* saponins 100 mg/kg	The AS rats	↓IL‐6, ↓MCP‐1	[45]
	Total *Panax notoginseng* saponin 5, 10 and 20 mg/kg	2F Fogarty balloon‐induced carotid artery injury model was established in rats	↓NF‐κBp65, ↓IL‐1β, ↓TNF‐α	[46]
	*Protopanaxadiol* saponins 100 mg/kg	ApoE^−^/^−^ mouse model of atherosclerosis	↓NF‐κB ↓VCAM‐1、↓ICAM‐1 ↓IL‐1β	[47]
Regulate platelet signalling pathway	Ginsenoside‐Rp1 10–20 μM	Inhibitory effects on ADP‐induced platelet aggregation	↓ATP, ↓P‐selectin, ↓Ca^2+^, ↓αIIbβ3, ↓p38 MAPK, ↓ERK2 activation	[48]
	*Panax notoginseng* saponins 200 mg/kg	Human umbilical vein endothelial cell injury and endothelial platelet adhesion	↓p‐p38MAPK, ↓p‐ERK1/2	[49]

### 4.1 Saponin Natural Products Interact With Platelet Membrane Glycoprotein Receptors

After PCI, vascular endothelial injury leads to collagen exposure, and plasma vWF specifically binds to platelet membrane glycoprotein GPIb‐IX‐V complex through its A1 domain. The GPIbα subunit of this complex strongly anchors to the vWF‐A1 domain under high shear conditions through its N‐terminal leucine rich repeat sequence, activating the PLCγ2/PKC signalling pathway and triggering platelet activation. This process not only promotes platelet adhesion and aggregation, but also lays the foundation for subsequent αIIbβ3 integrin dependent aggregation [50, 51]. Research has shown that *Panax notoginseng* saponins (PNS) can enhance antiplatelet aggregation and antithrombotic ability by regulating GPIbα subunits, inhibiting vWF mediated platelet adhesion [52, 53]. Shin et al. [40] found that Ginsenoside Ro dose‐dependently inhibited thrombin‐induced platelet aggregation and attenuated the binding of fibrinogen to αIIb/β3 by phosphorylating cyclic adenosine monophosphate (cAMP)‐dependently vasodilator‐stimulated phosphoprotein (VASP; Ser157). It was confirmed that PNS can inhibit shear‐induced calcium influx mediated by platelet Piezo1, thereby affecting the binding of platelet GPIbα to vWF, reducing platelet αIIbβ3 activation and vWF release, and ultimately inhibiting high shear‐induced platelet activation and aggregation [53]. Therefore, by targeting the binding domain of vWF and GPIb receptors, platelet adhesion and thrombosis can be effectively inhibited, laying the foundation for preventing and treating the occurrence of subsequent ISR.

### 4.2 Saponin Natural Products Inhibit Secretion of Platelet Adhesion Receptor P‐Selectin

Platelet selectin (P‐selectin) is an important adhesion molecule expressed after platelet activation and a crucial bridging molecule for intercellular interactions that is involved in platelet‐induced inflammation and ISR development [54]. Under normal circumstances, P‐selectin is stored in α‐granules and released on the surface of platelets upon stimulation. This rapid upregulation of P‐selectin expression allows P‐selectin to play a role in the early stages of platelet activation and endothelial cell dysfunction [55]. Once endothelial cells and platelets are activated, P‐selectin is upregulated and mediates the effects of platelets and white blood cells on endothelial cells, jointly constructing complex thrombus structures. Activated platelets bind firmly to damaged endothelial cells by binding to PSGL‐1 on the surface of endothelial cells through P‐selectin expressed on the surface. Oral administration of PNS significantly reduces vWF antigen in plasma and significantly decreases platelet aggregation rates induced by collagen, adrenaline, ADP and arachidonic acid (AA). P‐selectin also decreases significantly, which can inhibit platelet aggregation and protect the vascular endothelium. According to data from an in vitro experiment, after consuming 3 g of *Panax notoginseng* orally daily for 5 consecutive days, the levels of vWF and P‐selectin, which can play a haemostatic role, were detected to decrease simultaneously in plasma of volunteers, indicating a decrease in platelet adhesion and release ability after activation [41]. The interaction between platelets and endothelial cells can also activate signalling pathways within endothelial cells, leading to increased expression and release of inflammatory factors such as IL‐6, IL‐8 and MCP‐1, further exacerbating the inflammatory response. Continuous inflammatory stimulation can inhibit the repair and regeneration ability of endothelial cells while promoting the proliferation and migration of VSMCs, ultimately leading to intimal hyperplasia and the occurrence of ISR.

The interaction between platelets and white blood cells mediated by P‐selectin also plays a crucial role in the occurrence and development of ISR. Activated platelets bind to PSGL‐1 on the surface of white blood cells through P‐selectin, forming platelet and white blood cell aggregates. The formation of such aggregates not only promotes the adhesion and infiltration of white blood cells at the site of vascular injury but also activates white blood cells, releasing a large amount of inflammatory mediators to further activate endothelial cells and VSMCs, upregulate the expression of their surface adhesion molecules, promote more white blood cells and platelets to adhere and aggregate, form a vicious cycle, and accelerate the ISR. Therefore, targeting and antagonising P‐selectin can not only reduce platelet aggregation but also prevent the inflammatory response caused by the interaction between platelets and endothelial cells and between platelets and white blood cells to alleviate subsequent vascular obstruction. This may be an attractive antiplatelet strategy, thereby reducing the risk of ISR.

### 4.3 Saponin Natural Products Reduce the Release of Platelet Particles

Saponin natural products can inhibit platelet activation by blocking the release of platelet particles. The release of platelet particles is a crucial step in the process of platelet activation. Among them, Ca^2+^ is an important regulatory factor for platelet activation and granule release. Ca^2+^ is released from the pool stored in the endoplasmic reticulum into the cytoplasm, and this transient increase in Ca^2+^ concentration is a key signal for platelet activation. Calcium channel blockers and calmodulin inhibitors can reduce the intracellular Ca^2+^ concentration, thereby reducing platelet activation. Coincidentally, research has shown that *Panax notoginsengs* can inhibit the release of Ca^2+^ and TXA_2_ through the AA metabolism pathway, thereby suppressing platelet aggregation and activation [56]. Coincidentally, Zhang et al. [42] has shown that Ginsenoside Rb1 (0.01–10 μmol/L) can reduce the extracellular Ca^2+^ influx caused by thrombin by blocking the calcium channels regulated by receptors on the platelet membrane or directly inhibiting the release of Ca^2+^ stored in the cell, thereby reducing the intracellular Ca^2+^ concentration in platelets and exerting anticoagulant and antiplatelet effects. In addition, administering 500 mg/kg of *Panax notoginseng* extract by gavage to rats has a similar effect to aspirin, which can prolong the bleeding time of rats, significantly prolong coagulation indicators such as activated partial thromboplastin time, thrombin time, and plasma prothrombin time [43]. The mechanism may be that *Panax notoginseng* extract can reduce clotting factors such as Ca^2+^ and fibrinogen in the blood, improving coagulation function.

CD40L, also known as the CD40 ligand or CD154, is a type of transmembrane glycoprotein distributed on immune cells such as T lymphocytes and platelets [57]. CD40L is stored in resting platelets and rapidly released onto the membrane surface after platelet activation, where it is cleaved by extracellular proteases to form sCD40L. Over 95% of sCD40L in the circulation is derived from platelets [58]. When endothelial function is impaired, CD40 expression increases and, by binding to CD40L, activates the inflammatory response, activates platelets, and initiates endothelial dysfunction [59]. Elevated CD40L levels are associated with an increased risk of adverse cardiovascular events, such as myocardial infarction and stroke. A retrospective study revealed that increased expression of plasma sCD40L in patients with hyperlipidemia, diabetes and acute coronary syndrome (ACS) can lead to angioplasty and ISR [60, 61]. Therefore, by regulating the release of platelet particles, it is possible to effectively control thrombus formation and reduce the incidence of ISR without completely inhibiting platelet function.

### 4.4 Saponin Natural Products Inhibit Platelet Metabolic Pathways

Saponin natural products can inhibit platelet aggregation and activation by affecting metabolic pathways, thereby reducing the risk of thrombosis. PNS can inhibit the release of Ca^2+^ and TXA_2_ through the AA metabolism pathway, thereby suppressing platelet aggregation and activation [56]. In the experiment of observing the changes in platelet activity and gastric mucosal damage caused by the action of PNS and aspirin on acute myocardial infarction rat models, PNS can enhance the antiplatelet effect of aspirin and reduce the platelet aggregation rate. Its mechanism may be related to the regulation of the platelet AA metabolism pathway COX‐1 and cytochrome P450 (CYP) metabolism pathway by PNS [44]. The conclusion also indicates that PNS can alleviate the gastric mucosa damage caused by aspirin, improve gastrointestinal symptoms, and increase serum levels of motilin and gastrin by regulating the prostaglandin E2 metabolism pathway in gastric mucosa.

### 4.5 Saponin Natural Products Inhibit Release of Inflammatory Mediators

When blood vessels are damaged after stent implantation, the functional integrity of endothelial cells is disrupted, and the underlying collagen fibres are exposed to the vascular lumen. This change serves as a key initiating signal, rapidly recruiting inflammatory cells such as neutrophils and monocytes to the site of injury, forming a local inflammatory microenvironment. Inflammatory mediators (IL‐1, IL‐6, TNF‐α, MCP‐1, etc.) not only promote SMC proliferation, but also activate platelets. The release of factors such as PDGF and TGF‐β after platelet activation will further stimulate SMC proliferation, while platelet aggregation will form thrombi. During thrombus formation, infiltration of inflammatory cells will release more inflammatory mediators, forming a vicious cycle of ‘inflammation‐thrombus‐inflammation’ and accelerating the process of restenosis [35]. TNF‐α is an important pro‐inflammatory factor that can directly bind to platelet αIIbβ3 surface receptors, activate signalling pathways within platelets, and enhance platelet aggregation ability while NF‐κB is an important transcription factor that enters the nucleus after activation and regulates the expression of a series of genes related to platelet activation [62]. IL‐1β activates the NF‐κB signalling pathway after binding to the IL‐1 receptor on the surface of platelets. Studies showed that PNS not only inhibits the activation of NF‐κB and the expression of IL‐6 and MCP‐1 but also suppresses the expression of pro‐inflammatory factors, such as NF‐κBp65, IL‐1β, TNF‐α and calpain1 protein [45, 46]. *Protopanaxadiol* saponins (100 mg/kg) were found to inhibit integrin activation, adhesion molecule expression, and the release of inflammatory factors such as IL‐1β. Therefore, by inhibiting inflammatory factors and regulating platelet activation, PNS reduces the risk of thrombosis and thus delays the occurrence and development of ISR [47].

### 4.6 Saponin Natural Products Regulate Platelet Signalling Pathway

Saponin natural products exert antiplatelet effects by regulating intracellular signalling pathways. A study showed that Ginsenoside Rp1 inhibits ADP‐induced platelet aggregation by modulating the downstream signalling pathway of collagen receptor GPVI, which inhibits collagen‐stimulated platelet function. This effect involves stimulation of VASP and inhibition of ERK2 and p38‐MAPK [48]. The combination of 200 mg/kg PNS and dual antiplatelet drugs has a protective effect on human umbilical vein endothelial cells (HUVECs) and can improve endothelial platelet adhesion. The mechanism may be related to the protein content of p‐P38 MAPK and p‐ERK1/2 in the MAPA pathway of endothelial cells, effectively reducing the phosphorylation levels of p‐P38MAPK and p‐ERK1/2 [49].

### 4.7 Synergistic Actions of Saponin Natural Products in Regulating Various Pathways

Saponin natural products do not act on a single physiological link of platelets, but rather construct a multi‐level regulatory network against platelet aggregation, inflammation and proliferation by synergistically regulating multiple targets including platelet membrane glycoprotein receptor binding, granule secretion, metabolic pathways and inflammatory mediator release, thereby ultimately achieving an efficient effect in preventing and treating ISR.

Saponin natural products can simultaneously bind to platelet membrane glycoprotein receptors and inhibit the secretion of the adhesion receptor P‐selectin, forming a dual‐blockade network for platelet activation initiation and recruitment. On the one hand, their binding to GPIb can block the initial adhesion of platelets to vWF at the vascular endothelial injury site, preventing platelet anchoring and early aggregation from the source. On the other hand, inhibiting P‐selectin secretion can reduce the adhesive cross‐linking between activated platelets, neutrophils and vascular endothelial cells, thus cutting off the pro‐thrombotic and pro‐inflammatory crosstalk pathway of ‘platelet‐leukocyte‐endothelium’ [63]. The synergistic effect of these two aspects precisely inhibits the two core steps of platelet activation, lowering the probability of thrombosis initiation.

Furthermore, there is a close correlation between P‐selectin secretion and platelet granule release, and this characteristic serves as a key target for saponin natural products to achieve cascade inhibition. Inhibiting P‐selectin secretion not only directly blocks platelet adhesion and recruitment, but also synchronously down‐regulates the release of pro‐thrombotic and pro‐proliferative factors such as ADP and PDGF from granules. Conversely, reducing platelet granule release will further decrease the intracellular reserve of P‐selectin, fundamentally impairing its secretory capacity [64]. This positive feedback synergistic regulation not only enhances the anti‐platelet effect, but also alleviates vascular smooth muscle cell proliferation induced by granule‐derived factors, providing crucial support for the prevention and treatment of ISR.

## 5 Clinical Application of Saponin Natural Products in Antiplatelet Aggregation

Antiplatelet drugs, such as aspirin, clopidogrel and tirofiban, are commonly used for the prevention and treatment of cardiovascular and cerebrovascular diseases. However, the low reactivity of platelets to drugs and high risk of bleeding in some patients have prompted more research to focus on developing antithrombotic drugs from traditional Chinese medicine and natural medicines. Researchers discovered that various natural products have antiplatelet activity, among which saponins, terpenes and flavonoids are the main active substances studied [65].

Appropriate dual antiplatelet therapy is a key measure for preventing ISR. Although current dual antiplatelet therapy significantly reduces thrombotic events by inhibiting cyclooxygenase and P2Y12 receptors, the risk of bleeding cannot be ignored. The unique pharmacological activity of saponins and their synergistic effect with antithrombotic drugs have the potential to enhance efficacy and high safety. The use of PNS in patients after PCI can significantly reduce the likelihood of thrombosis and also alleviate bleeding caused by the use of dual antiplatelet drugs [66]. As some studies have confirmed, the combination of PNS and clopidogrel has increased antiplatelet activity, and its mechanism may be related to the inhibition of platelet aggregation, thereby achieving its pharmacological effect of improving microcirculation [67]. For example, Lu et al. [68]. studied on the efficacy of Compound Danshen Dripping Pills (composed of *Salvia miltiorrhiza* , *Panax notoginseng* and borneol) in improving aspirin resistance in post‐PCI patients. A total of 120 patients with aspirin resistance after PCI were enrolled. All patients had discontinued clopidogrel bisulfate for 18 months post‐PCI and were receiving aspirin monotherapy. The enrolled patients were additionally treated with Compound Danshen Dripping Pills for three months. The aspirin resistance was determined by the AA‐induced platelet aggregation rate. The changes in platelet aggregation rate before and after the additional administration of Compound Danshen Dripping Pills were compared. The results showed that the administration of Compound Danshen Dropping Pills effectively reduces the resistance of aspirin after PCI. Therefore, it is believed that Compound Danshen Dropping Pills can enhance patients' sensitivity to aspirin, and the two drugs have a synergistic antiplatelet effect.

The other clinical study observed the antithrombotic efficacy of Sanqi Tongshu Capsules (composed of PNS) combined with aspirin [69]. Fifty‐one elderly patients with CHD were randomly divided into a control group and an experimental group. The control group took aspirin orally alone, while the experimental group was orally administered Sanqi Tongshu Capsules (with PNS as the main component) combined with aspirin. After 8 weeks, the effects of the two groups on thromboelastography and the platelet inhibition rate via the AA‐induced pathway were observed. The experimental results showed that the combination of Sanqi Tongshu Capsules with aspirin has a significant antithrombotic effect. In terms of efficacy indicators, the combination therapy group had significantly better effects on *R* value, *K* value, Angle and MA value than aspirin alone, indicating that the combination therapy group had better antithrombotic efficacy than aspirin alone. The mechanism may be related to the fact that the combination therapy not only acts on platelets but also on the coagulation process involving coagulation factors and fibrinogen. Therefore, the combination of Sanqi Tongshu Capsules and aspirin has shown good clinical efficacy in the treatment of thrombosis, with no significant increase in the incidence of adverse reactions, which has clinical benefits for the occurrence and development of ISR.

## 6 Safety of Saponin Natural Products in Reducing Bleeding Risk

The core clinical dilemma of antithrombotic therapy lies in the inherent contradiction between antithrombotic efficacy and bleeding risk. Traditional antithrombotic agents (e.g., aspirin, clopidogrel) mostly achieve antithrombotic effects through potent inhibition of a single target, yet they tend to induce adverse reactions such as oral mucosal bleeding and gastrointestinal bleeding due to the excessive inhibition of physiological platelet function or impairment of vascular endothelial integrity. In contrast, saponin natural products, by virtue of their unique multi‐pathway synergistic regulatory mechanism, exert a potent antithrombotic effect while significantly reducing the bleeding tendency, thus demonstrating superior clinical safety.

Aspirin exerts an irreversible inhibitory effect on platelet aggregation. It permanently blocks the synthesis of TXA_2_ that promotes platelet aggregation and induces vascular constriction by irreversibly acetylating platelet COX‐1. This leads to long‐term impairment of platelet aggregation function and even affects physiological haemostasis (e.g., haemostasis after minor vascular injury of the gastrointestinal mucosa), thus rendering it prone to causing bleeding [70, 71]. However, most saponins exert reversible inhibition by only blocking the pathological platelet activation pathway without impairing the physiological haemostatic function of platelets. Studies have confirmed that *Panax notoginseng* triol saponins (PTS) can regulate GPIbα, but they act selectively on platelet aggregation under pathological conditions without interfering with the normal adhesion between vWF and GPIb during physiological haemostasis. The main mechanism is that PTS upregulates GPIbα expression and competitively inhibits GPIbα activity, thereby reducing vWF‐mediated platelet adhesion under pathological conditions and inhibiting platelet aggregation and thrombus formation [72].

The balance between TXA_2_ and prostacyclin (PGI_2_) is disrupted when platelets are activated, which promotes platelet aggregation. PTS acts to restore this balance by promoting PGI_2_ synthesis and moderately inhibiting TXA_2_ synthesis, rather than irreversibly blocking the key enzyme for TXA_2_ synthesis [72]. After PTS is metabolically cleared, the synthesis of TXA_2_ and PGI_2_ will return to physiological levels, and the signal transduction function of platelets will be restored accordingly. Ultimately, PTS achieves the unique property of ‘inhibiting pathological thrombosis while preserving physiological haemostasis’. In addition, regarding the adverse reactions such as oral mucosal and gastrointestinal bleeding associated with aspirin use, earlier studies have found that even the conventional minimum dose of aspirin (20 mg) can reduce TXA_2_ synthesis by over 90%. With the increase in dosage and concentration, it tends to cross the threshold of physiological haemostasis [73]. The antiplatelet effect of saponins is obviously dose‐dependent. At the effective antithrombotic dose, it only moderately inhibits platelet activation and does not reach the threshold of ‘haemostatic function loss’. PTS exerts a dose‐dependent (25–100 mg/kg) inhibitory effect on platelet aggregation and thrombosis in rats with middle cerebral artery occlusion (MCAO) model, with the smallest cerebral infarction area observed in the high‐dose group. PTS restores the balance by increasing PGI_2_ and decreasing TXA_2_, which is in sharp contrast to aspirin that irreversibly inhibits COX‐1, resulting in a significant reduction (over 90%) in TXA_2_ synthesis [72].

Studies have shown that after administration of aspirin (15.62 mg/kg), the levels of thromboxane B_2_ (TXB_2_) and the TXB2/6‐keto‐PGF1α ratio in the body significantly decrease. This inhibitory effect is also enhanced when combined with PNS (31.25 mg/kg). Especially from the downregulation effect of PNS combined with aspirin on the TXB2/6‐keto‐PGF1α ratio in healthy rats, it can be seen that PNS synergistically enhances the antiplatelet effect of aspirin, which may be achieved by inhibiting esterase‐mediated hydrolysis activity and increasing the exposure level of aspirin in vivo [74]. When using PNS in combination with aspirin, no increase in the potential risk of bleeding caused by the latter was found. In the combination therapy plan, when *Panax notoginseng* preparations are used in combination with Western medicine (aspirin, clopidogrel), a synergistic effect on reducing platelet aggregation rate and plasma TXB_2_ was observed without increases in the risk of bleeding. The results indicate that the regular combination of *Panax notoginseng* preparations with Western medicine can increase efficacy and safety [75]. Therefore, the application of natural saponins in antiplatelet therapy can improve its efficacy and safety.

## 7 Conclusion

During the development of ISR, saponin natural products inhibit platelet activation and the vicious cycle of ‘thrombosis inflammation’. The safety and sustainability advantages of saponin natural products are beneficial for providing a new strategy for ISR prevention and treatment. To extend clinical applications of saponin natural products for the prevention and treatment of ISR, a deeper understanding of the mechanism by which saponin natural products regulate platelet function is essential to prevent the occurrence and development of ISR.

## Author Contributions

L.Z. and X.L. wrote the manuscript and drew the figures. C.L., Y.Z., Z.L., H.S. searched the references and investigated clinical cases. All authors have read and approved the article.

## Funding

This work was supported by grants from the Natural Science Foundation of China (82470483 to L.Z.).

## Conflicts of Interest

The authors declare no conflicts of interest.

## Data Availability

The authors have nothing to report.

## References

[1] G. A. Roth , G. A. Mensah , C. O. Johnson , et al., “Global Burden of Cardiovascular Diseases and Risk Factors, 1990–2019,” Journal of the American College of Cardiology 76, no. 25 (2020): 2982–3021, 10.1016/j.jacc.2020.11.010. 33309175 PMC7755038

[2] S. Naniwa , S. Tsuda , G. Nakazawa , and S. Yamada , “Effectiveness of Directional Coronary Atherectomy in Treating Recurrent In‐Stent Restenosis: A Case Report,” European Heart Journal 8, no. 8 (2024): ytae233, 10.1093/ehjcr/ytae233. 39176022 PMC11339653

[3] A. Sakamoto , Y. Sato , R. Kawakami , et al., “Risk Prediction of In‐Stent Restenosis Among Patients With Coronary Drug‐Eluting Stents: Current Clinical Approaches and Challenges,” Expert Review of Cardiovascular Therapy 19, no. 9 (2021): 801–816, 10.1080/14779072.2021.1856657. 33470872

[4] I. D. Moussa , D. Mohananey , J. Saucedo , et al., “Trends and Outcomes of Restenosis After Coronary Stent Implantation in the United States,” Journal of the American College of Cardiology 76, no. 13 (2020): 1521–1531, 10.1016/j.jacc.2020.08.002. 32972528

[5] W. Sun , J. Zheng , and Y. Gao , “Targeting Platelet Activation in Abdominal Aortic Aneurysm: Current Knowledge and Perspectives,” Biomolecules 12, no. 2 (2022): 206, 10.3390/biom12020206. 35204706 PMC8961578

[6] S. G. Sparg , M. E. Light , and J. van Staden , “Biological Activities and Distribution of Plant Saponins,” Journal of Ethnopharmacology 94, no. 2–3 (2004): 219–243, 10.1016/j.jep.2004.05.016. 15325725

[7] D. Ji , Z. Y. Huang , C. H. Fei , W. W. Xue , and T. L. Lu , “Comprehensive Profiling and Characterization of Chemical Constituents of Rhizome of Anemarrhena Asphodeloides Bge,” Journal of Chromatography. B, Analytical Technologies in the Biomedical and Life Sciences 1060 (2017): 355–366, 10.1016/j.jchromb.2017.06.032. 28666227

[8] S. Bandopadhyay , U. Anand , V. S. Gadekar , et al., “Dioscin: A Review on Pharmacological Properties and Therapeutic Values,” BioFactors 48, no. 1 (2022): 22–55, 10.1002/biof.1815. 34919768

[9] Z. M. Thu , S. M. Oo , T. M. Nwe , et al., “Structures and Bioactivities of Steroidal Saponins Isolated From the Genera Dracaena and Sansevieria,” Molecules 26, no. 7 (2021): 1916, 10.3390/molecules26071916. 33805482 PMC8037284

[10] Z. Y. Shi , J. Z. Zeng , and A. S. T. Wong , “Chemical Structures and Pharmacological Profiles of Ginseng Saponins,” Molecules 24, no. 13 (2019): 2443, 10.3390/molecules24132443. 31277214 PMC6651355

[11] X. Y. Liu , S. Wang , C. J. Li , et al., “Dammarane‐Type Saponins From the Leaves of Panax Notoginseng and Their Neuroprotective Effects on Damaged SH‐SY5Y Cells,” Phytochemistry 145 (2018): 10–17, 10.1016/j.phytochem.2017.09.020. 29035776

[12] W. Z. Yang , Y. Hu , W. Y. Wu , M. Ye , and D. A. Guo , “Saponins in the Genus Panax L. (Araliaceae): A Systematic Review of Their Chemical Diversity,” Phytochemistry 106 (2014): 7–24, 10.1016/j.phytochem.2014.07.012. 25108743

[13] T. Wang , R. Guo , G. Zhou , et al., “Traditional Uses, Botany, Phytochemistry, Pharmacology and Toxicology of Panax Notoginseng (Burk.) F.H. Chen: A Review,” Journal of Ethnopharmacology 188 (2016): 234–258, 10.1016/j.jep.2016.05.005. 27154405

[14] J. M. Augustin , V. Kuzina , S. B. Andersen , and S. Bak , “Molecular Activities, Biosynthesis and Evolution of Triterpenoid Saponins,” Phytochemistry 72, no. 6 (2011): 435–457, 10.1016/j.phytochem.2011.01.015. 21333312

[15] G. Francis , Z. Kerem , H. P. S. Makkar , and K. Becker , “Reflections on ‘The Biological Action of Saponins in Animal Systems: A Review’,” British Journal of Nutrition 127, no. 7 (2022): 1034–1036, 10.1017/s0007114521004852. 34913419

[16] J. Su , Q. Su , S. Hu , X. Ruan , and S. Ouyang , “Research Progress on the Anti‐Aging Potential of the Active Components of Ginseng,” Nutrients 15, no. 15 (2023): 3286, 10.3390/nu15153286. 37571224 PMC10421173

[17] J. Kim , Y. Zhu , S. Chen , et al., “Anti‐Glioma Effect of Ginseng‐Derived Exosomes‐Like Nanoparticles by Active Blood‐Brain‐Barrier Penetration and Tumor Microenvironment Modulation,” Journal of Nanobiotechnology 21, no. 1 (2023): 253, 10.1186/s12951-023-02006-x. 37542285 PMC10401762

[18] Z. Zhang , Z. Song , F. Shen , et al., “Ginsenoside Rg1 Prevents PTSD‐Like Behaviors in Mice Through Promoting Synaptic Proteins, Reducing Kir4.1 and TNF‐α in the Hippocampus,” Molecular Neurobiology 58, no. 4 (2021): 1550–1563, 10.1007/s12035-020-02213-9. 33215390 PMC7676862

[19] B. K. Park , K. S. So , H. J. Ko , et al., “Therapeutic Potential of the Rhizomes of Anemarrhena Asphodeloides and Timosaponin A‐III in an Animal Model of Lipopolysaccharide‐Induced Lung Inflammation,” Biomolecules & Therapeutics 26, no. 6 (2018): 553–559, 10.4062/biomolther.2017.249. 29925223 PMC6254648

[20] R. A. Cao , R. Ji , M. Tabarsa , et al., “Purification, Characterization and Immunostimulatory Effects of Polysaccharides From Anemarrhena Asphodeloides Rhizomes,” International Journal of Biological Macromolecules 172 (2021): 550–559, 10.1016/j.ijbiomac.2021.01.088. 33465362

[21] X. Zhao , C. Liu , Y. Qi , et al., “Timosaponin B‐II Ameliorates Scopolamine‐Induced Cognition Deficits by Attenuating Acetylcholinesterase Activity and Brain Oxidative Damage in Mice,” Metabolic Brain Disease 31, no. 6 (2016): 1455–1461, 10.1007/s11011-016-9877-z. 27444169

[22] S. He , J. Yang , S. Hong , et al., “Dioscin Promotes Prostate Cancer Cell Apoptosis and Inhibits Cell Invasion by Increasing SHP1 Phosphorylation and Suppressing the Subsequent MAPK Signaling Pathway,” Frontiers in Pharmacology 11 (2020): 1099, 10.3389/fphar.2020.01099. 32792945 PMC7394018

[23] P. Zhang , X. Lei , L. Ou , et al., “Dioscin Ameliorates Silica‐Aggravated Systemic Lupus Erythematosus via Suppressing Apoptosis and Improving LC3‐Associated Phagocytosis in MRL/Lpr Mice,” International Immunopharmacology 116 (2023): 109814, 10.1016/j.intimp.2023.109814. 36773568

[24] J. Han , G. Shi , W. Li , Y. Xie , F. Li , and D. Jiang , “Preventive Effect of Dioscin Against Monosodium Urate‐Mediated Gouty Arthritis Through Inhibiting Inflammasome NLRP3 and TLR4/NF‐κB Signaling Pathway Activation: An in Vivo and in Vitro Study,” Journal of Natural Medicines 75, no. 1 (2021): 37–47, 10.1007/s11418-020-01440-7. 32761488

[25] J. O. Lee , E. Choi , K. K. Shin , et al., “Compound K, a Ginsenoside Metabolite, Plays an Antiinflammatory Role in Macrophages by Targeting the AKT1‐Mediated Signaling Pathway,” Journal of Ginseng Research 43, no. 1 (2019): 154–160, 10.1016/j.jgr.2018.10.003. 30662304 PMC6323178

[26] H. Xiao , S. Liu , B. Fang , et al., “Panax Notoginseng Saponins Promotes Angiogenesis After Cerebral Ischemia‐Reperfusion Injury,” Journal of Ginseng Research 48, no. 6 (2024): 592–602, 10.1016/j.jgr.2024.08.004. 39583172 PMC11584196

[27] X. Cui , L. Zhang , L. Lin , et al., “Notoginsenoside R1‐Protocatechuic Aldehyde Reduces Vascular Inflammation and Calcification Through Increasing the Release of Nitric Oxide to Inhibit TGFβR1‐YAP/TAZ Pathway in Vascular Smooth Muscle Cells,” International Immunopharmacology 143, no. Pt 3 (2024): 113574, 10.1016/j.intimp.2024.113574. 39520961

[28] J. D. Wang , Y. Cui , J. G. Wang , et al., “Ginseng Saponin Rh3 Pretreatment to Protect Myocardial Ischemia‐Reperfusion Injury in Rats,” Chinese Archives of Traditional Chinese Medicine 35, no. 11 (2017): 2783–2786, 10.13193/j.issn.1673-7717.2017.11.015.

[29] Y. Yu , J. Sun , J. Liu , P. Wang , and C. Wang , “Ginsenoside re Preserves Cardiac Function and Ameliorates Left Ventricular Remodeling in a Rat Model of Myocardial Infarction,” Journal of Cardiovascular Pharmacology 75, no. 1 (2020): 91–97, 10.1097/fjc.0000000000000752. 31599782

[30] X. Y. Deng , J. J. Chen , H. Y. Li , Z. Q. Ma , S. P. Ma , and Q. Fu , “Cardioprotective Effects of Timosaponin B II From Anemarrhenae Asphodeloides Bge on Isoproterenol‐Induced Myocardial Infarction in Rats,” Chemico‐Biological Interactions 240 (2015): 22–28, 10.1016/j.cbi.2015.08.001. 26277537

[31] N. Xing , Y. Wang , W. Wang , et al., “Cardioprotective Effect Exerted by Timosaponin BII Through the Regulation of Endoplasmic Stress‐Induced Apoptosis,” Phytomedicine 78 (2020): 153288, 10.1016/j.phymed.2020.153288. 32782218

[32] T. Liu , F. Yang , J. Liu , et al., “Astragaloside IV Reduces Cardiomyocyte Apoptosis in a Murine Model of Coxsackievirus B3‐Induced Viral Myocarditis,” Experimental Animals 68, no. 4 (2019): 549–558, 10.1538/expanim.19-0037. 31243190 PMC6842797

[33] M. V. Hjortbak , K. K. W. Olesen , J. M. Seefeldt , et al., “Translation of Experimental Cardioprotective Capability of P2Y(12) Inhibitors Into Clinical Outcome in Patients With ST‐Elevation Myocardial Infarction,” Basic Research in Cardiology 116, no. 1 (2021): 36, 10.1007/s00395-021-00870-y. 34037861

[34] J. Aoki and K. Tanabe , “Mechanisms of Drug‐Eluting Stent Restenosis,” Cardiovascular Intervention and Therapeutics 36, no. 1 (2021): 23–29, 10.1007/s12928-020-00734-7. 33222019

[35] S. F. Mause , E. Ritzel , A. Deck , F. Vogt , and E. A. Liehn , “Endothelial Progenitor Cells Modulate the Phenotype of Smooth Muscle Cells and Increase Their Neointimal Accumulation Following Vascular Injury,” Thrombosis and Haemostasis 122, no. 3 (2022): 456–469, 10.1055/s-0041-1731663. 34214997

[36] M. Pepe , G. Napoli , E. Carulli , et al., “Autoimmune Diseases in Patients Undergoing Percutaneous Coronary Intervention: A Risk Factor for In‐Stent Restenosis?,” Atherosclerosis 333 (2021): 24–31, 10.1016/j.atherosclerosis.2021.08.007. 34418682

[37] N. ElMokhtari , S. Zschernitz , S. Sebens , G. Simon‐Herrmann , and D. Krüger , “Cardiac Release and Kinetics of Cytokines After Elective Bare Metal Coronary Stenting,” Journal of Thrombosis and Thrombolysis 30, no. 4 (2010): 391–397, 10.1007/s11239-010-0466-4. 20229266

[38] R. Januszek , J. Bil , N. Gilis‐Malinowska , et al., “Long‐Term Outcomes Following Drug‐Eluting Balloon or Thin‐Strut Drug‐Eluting Stents for Treatment of In‐Stent Restenosis Stratified by Duration of Dual Antiplatelet Therapy (DEB‐Dragon Registry),” Postępy w Kardiologii Interwencyjnej 18, no. 1 (2022): 14–26, 10.5114/aic.2022.115631. 35982740 PMC9199027

[39] S. Yoneda , S. Abe , T. Kanaya , et al., “Late‐Phase Inflammatory Response as a Feature of In‐Stent Restenosis After Drug‐Eluting Stent Implantation,” Coronary Artery Disease 24, no. 5 (2013): 368–373, 10.1097/MCA.0b013e32836222ec. 23744617

[40] J. H. Shin , H. W. Kwon , H. J. Cho , M. H. Rhee , and H. J. Park , “Vasodilator‐Stimulated Phosphoprotein‐Phosphorylation by Ginsenoside Ro Inhibits Fibrinogen Binding to αIIb/β(3) in Thrombin‐Induced Human Platelets,” Journal of Ginseng Research 40, no. 4 (2016): 359–365, 10.1016/j.jgr.2015.11.003. 27746688 PMC5052406

[41] X. Y. Zhu , Q. Wang , B. X. Zhao , Y. Y. Song , W. Zhang , and Y. M. Zhao , “Study on Antiplatelet and Anticoagulant Mechanism of Sanqi,” Western Journal of Traditional Chinese Medicine 34, no. 8 (2021): 21–24.

[42] X. W. Zhang , X. Z. Lu , Y. X. Bao , and M. R. Rao , “Effects of Panax Notoginsenoside Rb1 Pretreatment on Free Calcium and Calcium Homeostasis in Hypoxia/Reoxygenation Injury Model in Neonatal Rat Hypertrophied Cardiomyocytes,” Gansu Medical Journal 31, no. 1 (2012): 4–7, 10.15975/j.cnki.gsyy.2012.01.013.

[43] A. J. Lau , D. F. Toh , T. K. Chua , Y. K. Pang , S. O. Woo , and H. L. Koh , “Antiplatelet and Anticoagulant Effects of *Panax notoginseng*: Comparison of Raw and Steamed *Panax notoginseng* With *Panax ginseng* and *Panax quinquefolium* ,” Journal of Ethnopharmacology 125, no. 3 (2009): 380–386, 10.1016/j.jep.2009.07.038. 19665534

[44] W. T. Wang , M. Xue , L. Yang , et al., “Experimental Research on the Protective Effect of *Panax notoginseng* Saponins on the Gastric Mucosa and the Anti‐Platelet Effect of Aspirin Based on Arachidonic Acid Metabolic Pathway,” Chinese Journal of Integrative Medicine on Cardio‐Cerebrovascular Disease 17, no. 9 (2019): 1315–1320.

[45] J. S. Fan , D. N. Liu , G. Huang , et al., “Panax Notoginseng Saponins Attenuate Atherosclerosis via Reciprocal Regulation of Lipid Metabolism and Inflammation by Inducing Liver X Receptor Alpha Expression,” Journal of Ethnopharmacology 142, no. 3 (2012): 732–738, 10.1016/j.jep.2012.05.053. 22683903

[46] Z. Yang , H. Zhang , M. An , et al., “Total Panax Notoginseng Saponin Inhibits Balloon Injury‐Induced Neointimal Hyperplasia in Rat Carotid Artery Models by Suppressing pERK/p38 MAPK Pathways,” Brazilian Journal of Medical and Biological Research 53, no. 1 (2020): e9085, 10.1590/1414-431x20199085. 31859914 PMC6915881

[47] C. Liu , R. Feng , J. Zou , F. Xia , and J. B. Wan , “20(S)‐protopanaxadiol Saponins Mainly Contribute to the Anti‐Atherogenic Effects of Panax Notoginseng in ApoE Deficient Mice,” Molecules 24, no. 20 (2019): 3723, 10.3390/molecules24203723. PMC6832312 31623159

[48] M. Endale , W. M. Lee , S. M. Kamruzzaman , et al., “Ginsenoside‐Rp1 Inhibits Platelet Activation and Thrombus Formation via Impaired Glycoprotein VI Signalling Pathway, Tyrosine Phosphorylation and MAPK Activation,” British Journal of Pharmacology 167, no. 1 (2012): 109–127, 10.1111/j.1476-5381.2012.01967.x. 22471932 PMC3448917

[49] J. J. Lv , Z. Wang , and E. Li , “Influence of *Panax notoginseng* Saponins Combined With Dual Antiplatelet Agents on Human Umbilical Vein Endothelial Cell Injury and Endothelial Platelet Adhesion,” Pharmacology and Clinics of Chinese Materia Medica 33, no. 1 (2017): 99–102, 10.13412/j.cnki.zyyl.2017.01.027.

[50] S. Goto , T. Hasebe , and S. Takagi , “Platelets: Small in Size but Essential in the Regulation of Vascular Homeostasis—Translation From Basic Science to Clinical Medicine,” Circulation Journal 79, no. 9 (2015): 1871–1881, 10.1253/circj.CJ-14-1434. 26278588

[51] X. R. Xu , D. Zhang , B. E. Oswald , et al., “Platelets Are Versatile Cells: New Discoveries in Hemostasis, Thrombosis, Immune Responses, Tumor Metastasis and Beyond,” Critical Reviews in Clinical Laboratory Sciences 53, no. 6 (2016): 409–430, 10.1080/10408363.2016.1200008. 27282765

[52] E. Mammadova‐Bach , T. Gudermann , and A. Braun , “Platelet Mechanotransduction: Regulatory Cross Talk Between Mechanosensitive Receptors and Calcium Channels,” Arteriosclerosis, Thrombosis, and Vascular Biology 43, no. 8 (2023): 1339–1348, 10.1161/atvbaha.123.318341. 37345523

[53] Y. L. Wang , J. Li , L. Liu , et al., “Biomechanopharmacological Study of Panax Notoginseng Saponins on High Shear‐Induced Platelet Aggregation and Thrombosis,” Chinese Journal of Experimental Traditional Medical Formulae 30, no. 23 (2024): 111–120, 10.13422/j.cnki.syfjx.20241018.

[54] M. Purdy , A. Obi , D. Myers , and T. Wakefield , “P‐ and E‐ Selectin in Venous Thrombosis and Non‐Venous Pathologies,” Journal of Thrombosis and Haemostasis 20, no. 5 (2022): 1056–1066, 10.1111/jth.15689. 35243742 PMC9314977

[55] U. Flierl , D. Fraccarollo , E. Lausenmeyer , et al., “Fractalkine Activates a Signal Transduction Pathway Similar to P2Y12 and Is Associated With Impaired Clopidogrel Responsiveness,” Arteriosclerosis, Thrombosis, and Vascular Biology 32, no. 8 (2012): 1832–1840, 10.1161/atvbaha.112.250720. 22652599

[56] Q. Yin , X. Zhang , S. Liao , X. Huang , C. C. Wan , and Y. Wang , “Potential Anticoagulant of Traditional Chinese Medicine and Novel Targets for Anticoagulant Drugs,” Phytomedicine 116 (2023): 154880, 10.1016/j.phymed.2023.154880. 37267694

[57] D. Lievens , A. Zernecke , T. Seijkens , et al., “Platelet CD40L Mediates Thrombotic and Inflammatory Processes in Atherosclerosis,” Blood 116, no. 20 (2010): 4317–4327, 10.1182/blood-2010-01-261206. 20705757 PMC2993630

[58] G. S. Hassan , Y. Merhi , and W. Mourad , “CD40 Ligand: A Neo‐Inflammatory Molecule in Vascular Diseases,” Immunobiology 217, no. 5 (2012): 521–532, 10.1016/j.imbio.2011.03.010. 21529993

[59] A. San Miguel Hernández , L. Inglada‐Galiana , R. García Iglesias , N. Alonso Castillejos , and F. J. Martín Gil , “Soluble CD40 Ligand: A Potential Marker of Cardiovascular Risk,” Revista Clínica Española 207, no. 8 (2007): 418–421, 10.1157/13108766. 17688874

[60] F. Angeli , P. Verdecchia , S. Savonitto , et al., “Soluble CD40 Ligand and Outcome in Patients With Coronary Artery Disease Undergoing Percutaneous Coronary Intervention,” Clinical Chemistry and Laboratory Medicine 60, no. 1 (2022): 118–126, 10.1515/cclm-2021-0817. 34714987

[61] M. S. Russell , A. Muralidharan , L. Larocque , et al., “Identification and Characterisation of the CD40‐Ligand of *Sigmodon hispidus* ,” PLoS One 13, no. 7 (2018): e0199067, 10.1371/journal.pone.0199067. 30052641 PMC6063397

[62] J. Huang , X. Li , X. Shi , et al., “Platelet Integrin αIIbβ3: Signal Transduction, Regulation, and Its Therapeutic Targeting,” Journal of Hematology & Oncology 12, no. 1 (2019): 26, 10.1186/s13045-019-0709-6. 30845955 PMC6407232

[63] M. Irfan , M. Kim , and M. H. Rhee , “Anti‐Platelet Role of Korean Ginseng and Ginsenosides in Cardiovascular Diseases,” Journal of Ginseng Research 44, no. 1 (2020): 24–32, 10.1016/j.jgr.2019.05.005. 32095094 PMC7033355

[64] X. Yang , X. Xiong , H. Wang , and J. Wang , “Protective Effects of *Panax notoginseng* Saponins on Cardiovascular Diseases: A Comprehensive Overview of Experimental Studies,” Evidence‐Based Complementary and Alternative Medicine 2014 (2014): 204840, 10.1155/2014/204840. 25152758 PMC4131460

[65] Y. Liu , A Cheminformatics Analysis of Natural Products From Different Biological Sources (Huazhong Agricultural University, 2020).

[66] X. Li , X. Xu , J. Xu , et al., “Study on the Quality Markers of *Panax notoginseng* Powder Based on Its Function of Promoting Blood Circulation,” Modernization of Traditional Chinese Medicine and Materia Medica‐World Science and Technology 24, no. 1 (2022): 19–34.

[67] Y. Hu and J. Wang , “Interactions Between Clopidogrel and Traditional Chinese Medicine,” Journal of Thrombosis and Thrombolysis 48, no. 3 (2019): 491–499, 10.1007/s11239-019-01945-3. 31471773

[68] Y. Lu and B. X. Xu , “Compound Danshen Dripping Pills Improves Clinical Efficacy in Post‐PCI Patients With Aspirin Resistance,” Chinese Journal of Integrative Medicine on Cardio‐Cerebrovascular Diseases 18, no. 12 (2020): 1919–1921.

[69] Q. F. Yang , D. Z. Cui , and X. Y. Yan , “Influence of Panax Notoginseng Saponins Combined With Aspirin on Senile Coronary Heart Disease by Thromboela‐Stogram,” Journal of Nanjing University of Traditional Chinese Medicine 32, no. 5 (2016): 425–427, 10.14148/j.issn.1672-0482.2016.0425.

[70] M. Hemler and W. E. Lands , “Purification of the Cyclooxygenase That Forms Prostaglandins. Demonstration of Two Forms of Iron in the Holoenzyme,” Journal of Biological Chemistry 251, no. 18 (1976): 5575–5579. 823151

[71] J. A. Giménez‐Bastida , W. E. Boeglin , O. Boutaud , M. G. Malkowski , and C. Schneider , “Residual Cyclooxygenase Activity of Aspirin‐Acetylated COX‐2 Forms 15 R‐Prostaglandins That Inhibit Platelet Aggregation,” FASEB Journal 33, no. 1 (2019): 1033–1041, 10.1096/fj.201801018R. 30096040 PMC6355089

[72] Z. Y. Xu , Y. Xu , X. F. Xie , et al., “Anti‐Platelet Aggregation of Panax Notoginseng Triol Saponins by Regulating GP1BA for Ischemic Stroke Therapy,” Chinese Medicine 16, no. 1 (2021): 12, 10.1186/s13020-021-00424-3. 33468191 PMC7816336

[73] B. B. Weksler , K. Tack‐Goldman , V. A. Subramanian , and W. A. Gay, Jr. , “Cumulative Inhibitory Effect of Low‐Dose Aspirin on Vascular Prostacyclin and Platelet Thromboxane Production in Patients With Atherosclerosis,” Circulation 71, no. 2 (1985): 332–340, 10.1161/01.cir.71.2.332. 3880671

[74] Z. X. Sun , Y. Wang , W. Q. Yang , Y. L. Wu , S. N. Hu , and S. Y. Du , “Research on the Anti‐Platelet Effects of Panax Notoginseng Saponins Combined With Aspirin Based on Molecular Docking and in Vivo Experiments,” Frontiers in Pharmaceutical Sciences 23, no. 12 (2020): 2317–2321+2394.

[75] Y. Li , F. W. Yang , M. Y. Zhang , et al., “Effect of Panax Notoginseng Preparations on Platelet Function,” China Journal of Chinese Materia Medica 42, no. 21 (2017): 4226–4233, 10.19540/j.cnki.cjcmm.20170901.004. 29271165

